# Innovative Multiparametric Characterization of Carotid Plaque Vulnerability by Ultrasound

**DOI:** 10.3389/fphys.2020.00157

**Published:** 2020-03-03

**Authors:** Guillaume Goudot, Lina Khider, Olivier Pedreira, Jonathan Poree, Pierre Julia, Jean-Marc Alsac, Kisaki Amemiya, Patrick Bruneval, Emmanuel Messas, Mathieu Pernot, Tristan Mirault

**Affiliations:** ^1^Physics for Medicine Paris, INSERM U1273, ESPCI Paris, CNRS FRE 2031, PSL Research University, Paris, France; ^2^Vascular Department, Hôpital Européen Georges Pompidou, APHP, Paris, France; ^3^INSERM U970 PARCC, Paris University, Paris, France

**Keywords:** carotid plaque, plaque vulnerability, ultrafast ultrasound imaging, wall shear stress, elastography

## Abstract

**Objective:**

The degree of stenosis of a carotid plaque is a well-established risk factor for ischemic stroke. Nevertheless, the risk of ipsilateral stroke in asymptomatic carotid stenosis remains low and new imaging markers are needed to better target which patients would benefit most from endarterectomy or intensive medical therapy. Ultrafast ultrasound imaging offers parameters helping at characterizing the carotid plaque by shear wave elastography and Ultrafast Doppler (UFD). We aimed at using these techniques to characterize 3 different ultrasound biomarkers: plaque stiffness heterogeneity, wall shear stress (WSS) and intraplaque micro-flows and to correlate these biomarkers with findings on computed tomography angiography (CTA) and the pathological examination.

**Methods:**

We present the case of a multimodal evaluation of a carotid plaque using ultrasound. Elastography has been coupled to the WSS assessment and the detection of intraplaque micro-flows by UFD. The data have been compared to CTA and to the pathology examination of the tissue after carotid endarterectomy.

**Results:**

Elastography allowed at identifying stiff areas corresponding to calcifications, as well as a soft area corresponding to an intraplaque hemorrhage. The flow evaluation with UFD showed an increase of the WSS along the plaque and identified the presence of a plaque rupture, confirmed by the pathologist.

**Conclusion:**

Ultrafast ultrasound imaging is an innovative, easily accessible technique that provides imaging modalities on top of the conventional B-mode. Ultrafast ultrasound biomarkers such as plaque stiffness heterogeneity, WSS and intraplaque micro-flows could help to define the vulnerability of the carotid plaque in order to stratify patients that could benefit most from endarterectomy or intensive medical therapy.

## Introduction

Carotid endarterectomy in case of asymptomatic plaque has been tested by 2 large randomized trials, ACAS and ACST trials (Walker et al., 1995; Halliday et al., 2010). A small but significant absolute risk reduction at 5 years of 5.9% (5.1 vs. 11.0% of ipsilateral stroke) and 5.4% (6.4 vs. 11.8% for any territory stroke) were respectively found. But these past studies overestimated the stroke risk using old standards medical intervention (Naylor, 2011; Abbott et al., 2017). With lower thresholds for defining hypertension and hypercholesterolemia, better blood pressure lowering drugs, statins and more people giving up smoking, the risk of ipsilateral stroke from a carotid plaque has been reduced to approximately 1% per year since ACAS and ACST trials (Naylor et al., 2009), now making the relevance of endarterectomy debatable (Abbott, 2009). There are, however, still patients having asymptomatic carotid plaques at “higher-than-average” risk of ipsilateral stroke. For example, as reported by Nicolaides et al. (2010), asymptomatic patients with a 90–99% ECST stenosis, a history of contralateral transient ischaemic attack or stroke and no discrete white areas and a grayscale median over 30, had a risk of ipsilateral stroke over 5 years of 13.3% when plaque area exceeded 80 mm^2^ (Nicolaides et al., 2010). Better risk stratification of people with carotid stenosis may mean better use of treatments to reduce the risk of ipsilateral stroke (as in the case of carotid procedural intervention) or all arterial disease complications (as in the case of medical intervention- lifestyle coaching and medications).

All persons with arterial disease should receive current optimal medical intervention to reduce the risk of stroke and other arterial disease complications using clinically identified risk factors (such as hypertension, hyperlipidaemia or smoking) for risk stratification. This is because current best evidence indicates a likely overall patient benefit using this approach. In addition, arterial imaging technology could be used to spare people from unnecessary and potentially harmful procedures or other treatments. It could be also used to better select persons for carotid procedures or particularly effective but risky or expensive medical treatments.

Biomarkers to identify plaques at higher risk of ipsilateral stroke have been proposed with different imaging modalities, magnetic resonance imaging (MRI), ultrasound, and computed tomography angiography (CTA) (Aboyans et al., 2017; Naylor et al., 2018; Saba et al., 2019). These biomarkers are based on comparisons with the histological profiles of vulnerable plaques, determined by the presence of a ruptured fibrous cap or an intra-plaque hemorrhage for instance (Lovett et al., 2004). Using ultrasound imaging, plaque vulnerability can be assessed by measuring plaque B-mode echogenicity (Geroulakos et al., 1993; Topakian et al., 2011), estimation of plaque volume (Nicolaides et al., 2010), stenosis progression (Kakkos et al., 2014), impaired vascular reserve (King et al., 2011) and spontaneous embolization on transcranial Doppler (Markus et al., 2010). The combination of combined markers would thus make it possible to identify the most at-risk plaques.

The aim of our research is to provide biomarkers with ultrafast ultrasound imaging that could easily be combined to identify patients who would benefit most from endarterectomy or intensive medical therapy. In order to maintain the benefit of such interventions, the objective would be to identify plaques with an annual risk of ipsilateral stroke over 3%. In our laboratory we developed specifically dedicated sequences for the evaluation of the carotid plaque by using ultrafast imaging modalities. We present here the multimodal evaluation of a patient at “higher-than-average” risk of ipsilateral stroke based on the stenosis progression and a large plaque area. The aim of this case is to show a proof of concept on the feasibility of ultrafast imaging evaluation by combining several methods of tissue and blood flow analysis.

## Case

**FIGURE 1:**
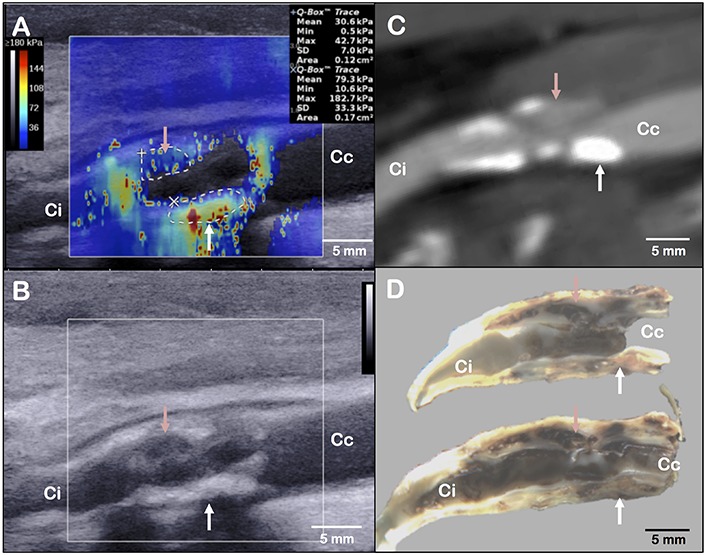
Layout of elastography assessment **(A)**, B-mode ultrasound acquisition **(B)**, computed tomography angiography **(C)** and gross examination **(D)** of the same plaque in a longitudinal axis view. The red arrow indicates the main haemorrhagic area on panel **(D)**. It corresponds to a low stiffness **(A)**, hypoechogenic **(B)** and low-density **(C)** area. The white arrow indicates a fibrous and calcified area on panel **(D)**. It corresponds to a high stiffness **(A)**, hyperechogenic **(B)** and high-density **(C)** area. Ci, internal carotid; Cc, common carotid.

A 68-year-old male with a medical history of hypertension and hyperlipidaemia was monitored every year by Doppler ultrasound for an atherosclerotic stenosis of the left carotid bifurcation. He was non-smoker, he received valsartan 160 mg, amlodipine 10 mg, pravastatin 40 mg and aspirin 75 mg daily. Still asymptomatic, without ischemic complication, the degree of stenosis increased up to 70% NASCET compared to 55% one year before. The plaque was large, at 84 mm^2^, with a grayscale median at 64 (no unit). A CTA confirmed the presence of a 70% NASCET carotid stenosis associated with calcifications (Figure 1). The patient was therefore referred for a carotid endarterectomy. After being informed of the risks of this procedure, the patient accepted the surgery and signed a written statement of consent. The UF-plaque study evaluating the usefulness of ultrafast ultrasound imaging (UF) multiple parameters to better define the plaque vulnerability was further presented to the patient. He signed the consent for inclusion and publication of this case report. We present below the results of this UF evaluation.

### Elastography Evaluation

**FIGURE 2:**
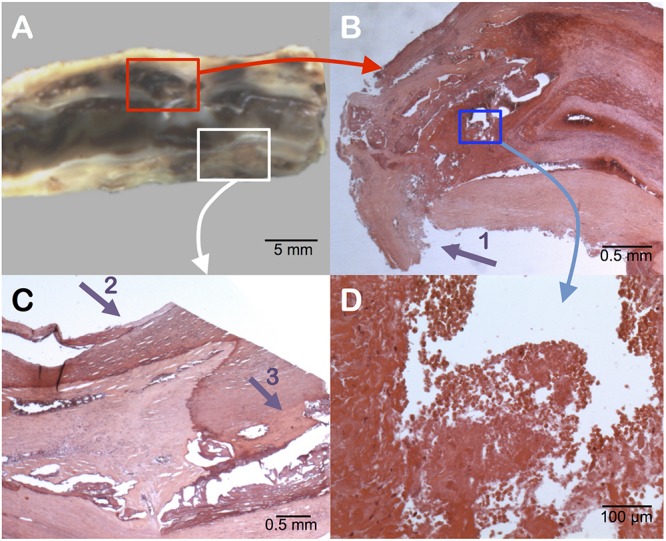
Pathology examination of the carotid plaque. Gross longitudinal section **(A)**. Presence of an intraplaque hemorrhage (surrounded by the red box) in the upper section, as seen in cross-sectional microscopic analysis **(B)**. Identification of the rupture zone of the fibrous cap (arrow 1). At higher magnification of the blue box **(D)** the intraplaque hemorrhage contains numerous red blood cells with intact membranes, confirming a recent hemorrhage. The posterior part of the plaque (white box) shows surface calcifications (arrow 2) and deep calcifications (arrow 3) **(C)**.

Shear wave elastography is an ultrasound-based technique for real-time and quantitative imaging of soft tissue viscoelastic properties. UF, using a frame rate of 5,000 frames per second, allows tracking transient mechanical shear waves generated by the ultrasonic probe through the acoustic propagation. The velocity of shear waves is directly linked to the tissue stiffness (Bercoff et al., 2004). The elastography mode, by the use of an Aixplorer^®^, ultrasound scanner (SuperSonic Imagine©, Aix-en-Provence, France) and a linear probe (SL10-2, central frequency of 7.5 MHz), provides stiffness mapping of the arterial wall superimposed to the B-mode image (Tanter and Fink, 2014). This technique can be applied to the analysis of carotid plaques (Figure 1) (Ramnarine et al., 2014; Garrard et al., 2015; Lou et al., 2017). In our case, we observed heterogeneity of the local elastic Young modulus, corresponding to the arterial wall stiffness, with the presence of a stiff area (area’s mean elasticity of 79.3 ± 33.3 kPa) in the posterior wall, and a low stiffness area (area’s mean elasticity of 30.6 ± 7.0 kPa) in the anterior wall. The posterior wall had scattered echoes with shadow artifacts in B-mode ultrasound that could correspond to the calcifications depicted on the CTA. The anterior wall had a hypoechogenic segment in B-mode ultrasound that could correspond to the low-density area on the CTA (Figure 1 and Supplementary Figure S1). While the stiff part of the plaque corresponded to surface and deep calcifications, the low-stiffness part of the plaque could correspond to the intraplaque hemorrhage or the necrotic lipid core located at the most stenotic segment found on gross examination (Figure 2).

### Ultrafast Doppler (UFD) Evaluation

**FIGURE 3:**
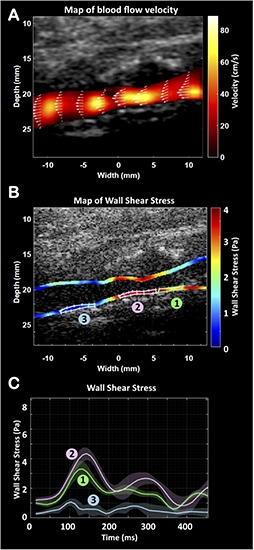
Ultrafast vector flow imaging. Representation of the velocities map **(A)**. Representation of the wall shear stress (WSS) map along the carotid walls **(B)** and the changes over the cardiac cycle **(C)** at the plaque’s ascent (1), plaque’s peak (2) and plaque’s descent (3).

Two UFD imaging sequences have been used here: the first sequence was designed for ultrafast vector flow imaging (angle-independent quantitative Doppler mode) and the second sequence was designed for ultra-sensitive Doppler imaging of low velocity blood flows. To map the flow dynamics correctly in the carotid artery, it is imperative to resolve the beam-flow angle dependence problem. Vector Doppler imaging can estimate flow velocities at each point of the lumen without any angle correction (Dort et al., 2012; Jensen et al., 2018), giving access to the wall shear stress (WSS) (Poelma et al., 2012; Leow and Tang, 2018; Goudot et al., 2019a, b): the tangential force exerted by the blood on the vessel wall. Vector Doppler imaging relies on Doppler estimation performed at different beam-flow angles, with a minimum of two angles, to obtain two independent frequency shift equations and derive the absolute velocity of each point. The ultrafast vector flow imaging used the same Aixplorer^®^ scanner, the same linear probe and a sequence of 3 plane waves [–10°, 0°, 10°] leading to a frame rate of 5,000 Hz. The WSS data were generated by computing the gradients of velocity in the vicinity of the arterial wall multiplied by the blood viscosity. In this case, the evaluation showed an increase of the WSS along the carotid plaque from 3.82 Pa at the plaque ascent, to a maximum of 4.71 Pa at the most stenotic segment (plaque’s peak) and dropped below 1.45 Pa at the plaque descent (Figure 3 and Supplementary Video S1).

**FIGURE 4:**
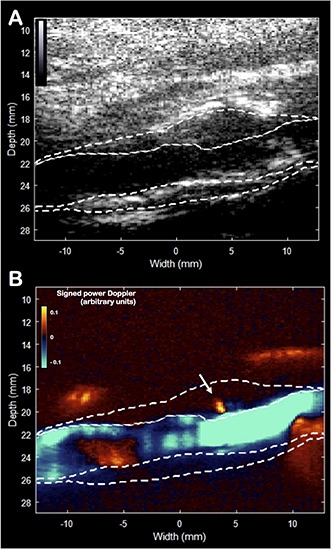
Ultra-sensitive Doppler imaging. B-mode picture **(A)** and the signed power Doppler picture **(B)**. The plaque was manually delineated. The white arrow indicates the presence of an isolated centrifugal micro-flux originating from the lumen inside the plaque’s wall, which may correspond to a rupture zone of the fibrous cap.

Unlike the vector flow imaging mode, ultra-sensitive Doppler mode has been developed to enhance the detection of low velocity flows with a high spatial resolution (Macé et al., 2011). Applied to the carotid artery, the objective was to detect micro-flows within the plaque. The sensitivity of detection is improved by using ultrafast Doppler imaging and plane wave compounding (Bercoff et al., 2011) combined with a spatio-temporal singular value decomposition clutter filter (Demené et al., 2015). The ultra-sensitive Doppler imaging used the same ultrasound scanner, the same probe and a sequence of 16 plane waves [–15°, –13°, –11°, –9°, –7°, –5°, –3°, –1°, 1°, 3°, 5°, 7°, 9°, 11°, 13°, 15°] leading to a frame rate of 950 Hz. Compared to conventional Doppler, the compounding of these 16 plane waves increases the sensitivity to 30 times (Macé et al., 2011) showing very small flow changes. In this case, the presence of a centrifugal micro-flow from the lumen was identified within the anterior wall (Figure 4 and Supplementary Video S1) and located in the area of soft stiffness described above. Ultra-sensitive Doppler allows to identify ascending and descending flows. During the cardiac cycle, the ascending flow occurs in systole, and the descending flow occurs in diastole, as shown in the movie. We believe that this flow, not observable in conventional Doppler technique, is the witness of an ulceration in the plaque’s anterior wall.

### Surgical Observation

During the intervention, the surgeon noticed the presence of a severe and calcified stenosis with a plaque ulceration. No complication, either immediately after surgery or after 1 year of follow-up, were noticed. Post-operative Duplex ultrasound revealed no residual stenosis.

### Pathology Examination

Gross examination of the removed plaque was assessed on a longitudinal section (Figure 2). It noticed the presence of a large intraplaque hemorrhage and a plaque rupture located in the anterior wall. Histology revealed diffuse microcalcifications and thick calcifications in the posterior wall. According to Lovett et al., the presence of a rupture of the fibrous cap and an intraplaque hemorrhage were significantly associated with irregularity of the carotid plaque on angiography, which appeared as a strong independent predictor of ipsilateral ischemic stroke on medical treatment at all degrees of stenosis (hazard ratio 1.80) (Rothwell et al., 2000), categorizing the plaque as vulnerable (Eliasziw et al., 1994; Streifler et al., 1994; Rothwell et al., 2000; Lovett et al., 2004).

## Discussion

This case illustrates a multiparametric assessment of a carotid plaque by UF. The vulnerability of the plaque was confirmed by the presence of a plaque ulceration and a large intraplaque hemorrhage described on gross examination. These characteristics had not been seen neither by Duplex ultrasound nor by CT scan. In addition to conventional B-mode, UF enables us to better identify structures by stiffness analysis and WSS measurement. In this case, UF identified a soft area with a low velocity flow disappearing in the anterior wall of the carotid artery. These data may attest the presence of a hemorrhage and ulceration in the plaque’s wall. The ultrafast vector flow imaging, recently developed, offers the unique opportunity to directly measure WSS by ultrasound. MRI previously tried, first by Computational Flow Dynamics and recently by 4D flow sequences with to address the direct assessment of the WSS. High WSS values were found associated with the presence of intraplaque hemorrhage (Tuenter et al., 2016), as we report in our case. Moreover, as reported by Slager et al. (2005) the WSS may play a role in the generation of rupture-prone vulnerable plaque. As the intraplaque hemorrhage is known to be associated with an increased risk of stroke in carotid plaques, presence of high WSS values could argue for a vulnerable plaque.

Lastly, the ultra-sensitive Doppler imaging aims at detecting micro-flows within tissue without the need for any contrast agents. This method can identify a flow irrigating into the plaque, which can indicate the presence of a plaque ulceration, a characteristic associated with an increased risk of embolic events (Eliasziw et al., 1994; Handa et al., 1995). The high frame rate (950 Hz) allows here to detect low velocity flows, not accessible with all conventional imaging techniques (ultrasound, CT and MRI) (Macé et al., 2011).

The main limitation regarding the parameters presented is related to the presentation of a single case. The sole objective was to illustrate the feasibility of these ultrafast imaging modalities and no conclusion on the validation of the parameters presented can be provided here. A prospective validation is indeed required to demonstrate the value of these markers.

## Conclusion

In conclusion, UF is an innovative technology that conveys, on top of a B-Mode imaging, a shear wave elastography mode and an ultrafast Doppler mode leading to new parameters to better characterize the carotid plaque by measuring stiffness, WSS and micro-flows. The UF–plaque study (NCT03234257) is currently on-going aiming at demonstrating the correlation between stiffness measurement, WSS, micro-flows detection with plaque vulnerability criteria determined by histology on a prospective cohort of patients with carotid endarterectomy. For the future, the local evaluation of the carotid plaque by UF could provide an opportunity to perform a multimodal analysis including clinical criteria (history of previous cerebral infarction or transient ischemic attack), and association with brain imaging criteria (silent infarction), in order to select patients at higher than average risk of stroke that would most benefit from carotid endarterectomy or intensive medical treatment.

## Data Availability Statement

All datasets generated for this study are included in the article/Supplementary Material.

## Ethics Statement

The studies involving human participants were reviewed and approved by the Comités de Protection des Personnes, CPP N° 2017T2-13. The patients/participants provided their written informed consent to participate in this study.

## Author Contributions

GG, LK, and TM wrote the manuscript. GG and EM performed the ultrasound analysis. PJ and J-MA performed the surgery. PB and KA performed the histological analysis. MP, OP, and JP performed the post-processing of the ultra-fast Doppler acquisitions. TM performed final approval of the version to be published and agreed to be accountable for all aspects of the work in ensuring that questions related to the accuracy and integrity of any part of the work are appropriately investigated and resolved.

## Conflict of Interest

The authors declare that the research was conducted in the absence of any commercial or financial relationships that could be construed as a potential conflict of interest.

## Supplementary Material

FIGURE S1 Carotid plaque contouring visualized by different imaging modalities: B-mode ultrasound **(A)**, shear wave elastography **(B)**, ultrasensitive Doppler **(C)**, computed tomography **(D)**. The plaque is contoured on gross analysis after a longitudinal section of the carotid bifurcation **(E)**. The areas of calcifications (gray) and intra-plaque hemorrhage are shown on the carotid stenosis figure **(F)**. Ci, internal carotid; Cc, common carotid.

VIDEO S1 Evaluation of blood flow by Ultrafast Doppler at the carotid plaque level over the cardiac cycle. Representation of flow velocity vectors (1), wall shear stress (2), and micro-flow obtained with the ultra-sensitive Doppler sequence (3).
