# Supplementary figures and images for: Innovative Multiparametric Characterization of Carotid Plaque Vulnerability by Ultrasound
Source: Front Physiol. 2020 Mar 3;11:157. doi: 10.3389/fphys.2020.00157 (PMC7064056; doi:10.3389/fphys.2020.00157)

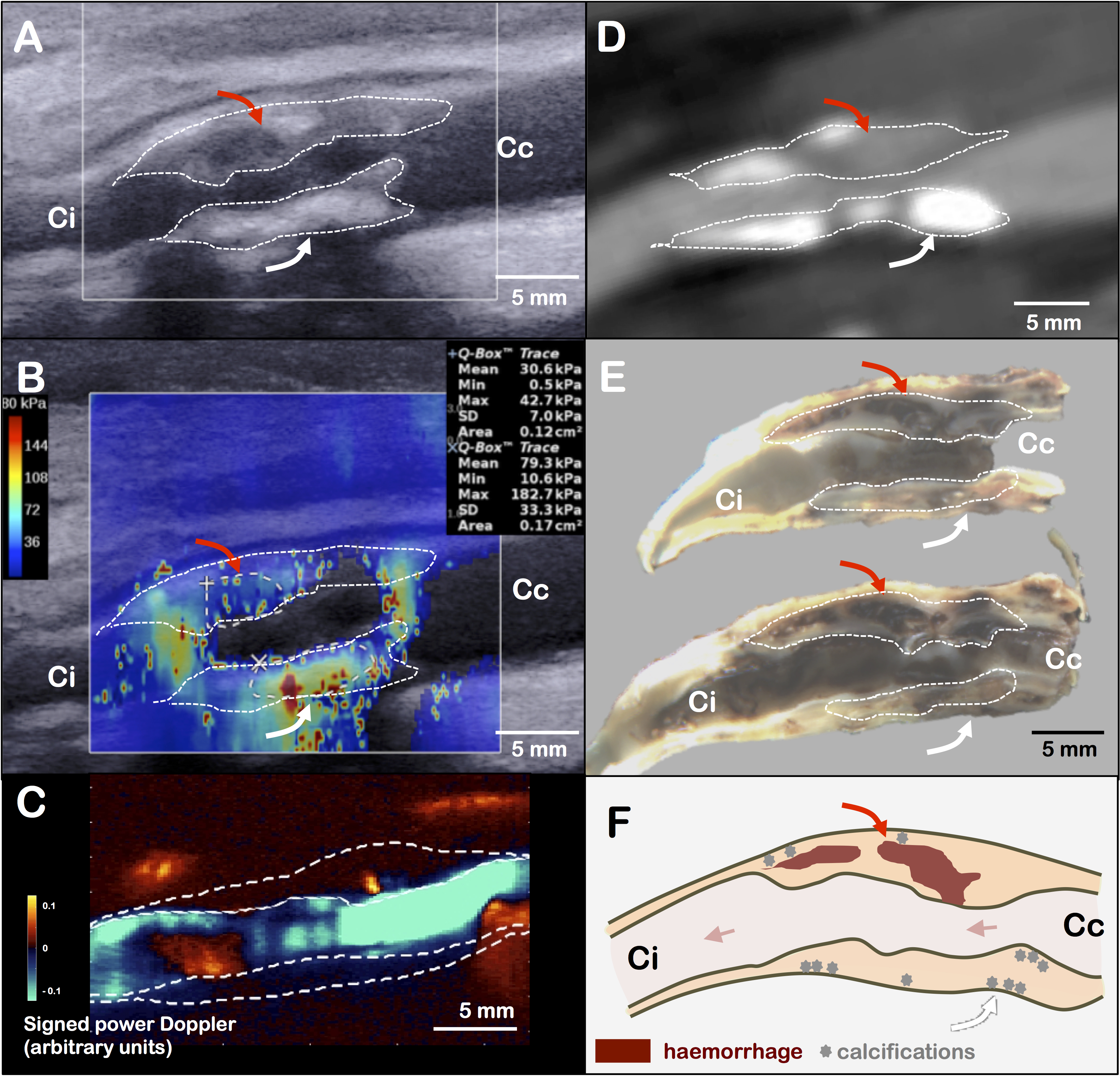

Supplement: FIGURE S1 — Carotid plaque contouring visualized by different imaging modalities: B-mode ultrasound (A), shear wave elastography (B), ultrasensitive Doppler (C), computed tomography (D). The plaque is contoured on gross analysis after a longitudinal section of the carotid bifurcation (E). The areas of calcifications (gray) and intra-plaque hemorrhage are shown on the carotid stenosis figure (F). Ci, internal carotid; Cc, common carotid. [file Image_1.JPEG]
